# Ethnobotany, diverse food uses, claimed health benefits and implications on conservation of barley landraces in North Eastern Ethiopia highlands

**DOI:** 10.1186/1746-4269-7-19

**Published:** 2011-06-28

**Authors:** Hailemichael Shewayrga, Peter A Sopade

**Affiliations:** 1 Sirinka Agricultural Research Center, P.O.Box 74, Woldia, Ethiopia; 2 School of Agriculture and Food Sciences, The University of Queensland, QLD 4072, Australia; 3 Centre for Nutrition and Food Sciences, The University of Queensland, St Lucia, QLD 4072, Australia

## Abstract

**Background:**

Barley is the number one food crop in the highland parts of North Eastern Ethiopia produced by subsistence farmers grown as landraces. Information on the ethnobotany, food utilization and maintenance of barley landraces is valuable to design and plan germplasm conservation strategies as well as to improve food utilization of barley.

**Methods:**

A study, involving field visits and household interviews, was conducted in three administrative zones. Eleven districts from the three zones, five *kebeles *in each district and five households from each *kebele *were visited to gather information on the ethnobotany, the utilization of barley and how barley end-uses influence the maintenance of landrace diversity.

**Results:**

According to farmers, barley is the "king of crops" and it is put for diverse uses with more than 20 types of barley dishes and beverages reportedly prepared in the study area. The products are prepared from either boiled/roasted whole grain, raw- and roasted-milled grain, or cracked grain as main, side, ceremonial, and recuperating dishes. The various barley traditional foods have perceived qualities and health benefits by the farmers. Fifteen diverse barley landraces were reported by farmers, and the ethnobotany of the landraces reflects key quantitative and qualitative traits. Some landraces that are preferred for their culinary qualities are being marginalized due to moisture shortage and soil degradation.

**Conclusions:**

Farmers' preference of different landraces for various end-use qualities is one of the important factors that affect the decision process of landraces maintenance, which in turn affect genetic diversity. Further studies on improving maintenance of landraces, developing suitable varieties and improving the food utilization of barley including processing techniques could contribute to food security of the area.

## Background

In developed countries, barley is primarily used for animal feed, malting and brewing with little designated for food. However, in Ethiopia and many developing countries, barley is produced mainly as a food crop, and it is the fifth most important cereal crop in Ethiopia after tef, maize, sorghum and wheat [1]. The country is recognized as the secondary centre of diversity for barley [2], and the Ethiopian barley germplasm has been important worldwide as a source of useful genes for traits such as disease resistance [3,4]. The crop is produced by subsistence farmers mostly grown as landraces with little or no application of fertilizers, pesticides and herbicides [5]. Landraces are defined as traditional varieties developed through natural and human selections, which are named and maintained by traditional farmers to meet their social, economic, cultural, and ecological needs [6]. Barley is cultivated from 1400 to over 4000 m above sea level, and its importance increases in drought-prone areas and at higher elevations (above 2800 m) where poor soil fertility, frost, water logging, and soil acidity and degradation are the major yield limiting factors [5,7]. The major barley producing regions in Ethiopia are Oromiya, Amhara and Tigray Regional States, which account for about 87% of the national barley production [1]. Therefore, barley holds an important position in the food security of Ethiopia.

Access to a range of crop genetic variability is critical to the success of breeding programs, and consequently to food security and human nutrition [8,9]. Landraces are considered more locally adapted and genetically variable than modern cultivars [6,10]. They contribute to agricultural production around the world, particularly for the rural poor in marginal environments as source of seed for next season planting [5,10]. Farmers make crop maintenance decisions based on combinations of factors including adaptability, yield, socio-cultural values and food traditions as well as nutritional values. These decisions affect the genetic diversity of crop populations [7,11,12]. Farmers' maintenance approaches have allowed the continual evolution of landraces diversity in their area of adaptation. This diversity has been the key to food security for generations and an invaluable resource for crop improvement activities around the world.

Knowledge of the utilization and traditional food processing techniques as well as types of germplasm maintained by farmers are prerequisite for investigating ways to improve the germplasm maintenance of a food crop. The information is also important for understanding nutritional qualities as well as processing techniques. In Ethiopia, Tsegaye and Berg [13] investigated the utilization of durum wheat landraces in East Shewa. They identified 14 dishes and two drinks derived from landraces. This richness in food tradition was associated with a high level of on-farm landrace diversity. With regards to barley, there have been efforts, though limited, on documentation of its utilization and ethno-botany for some parts (e.g. Central) of Ethiopia [5,14-16]. We conducted a study on barley utilization in North Eastern Ethiopia with the main objectives to (1) document the importance, ethnobotany and types of barley landraces grown; (2) investigate and gather information on the utilization of barley and its importance in the diets of the people, and the dishes prepared; (3) examine how barley end-uses influence the maintenance of its landraces.

## Research Methods

### Description of the study area

**Figure 1:**
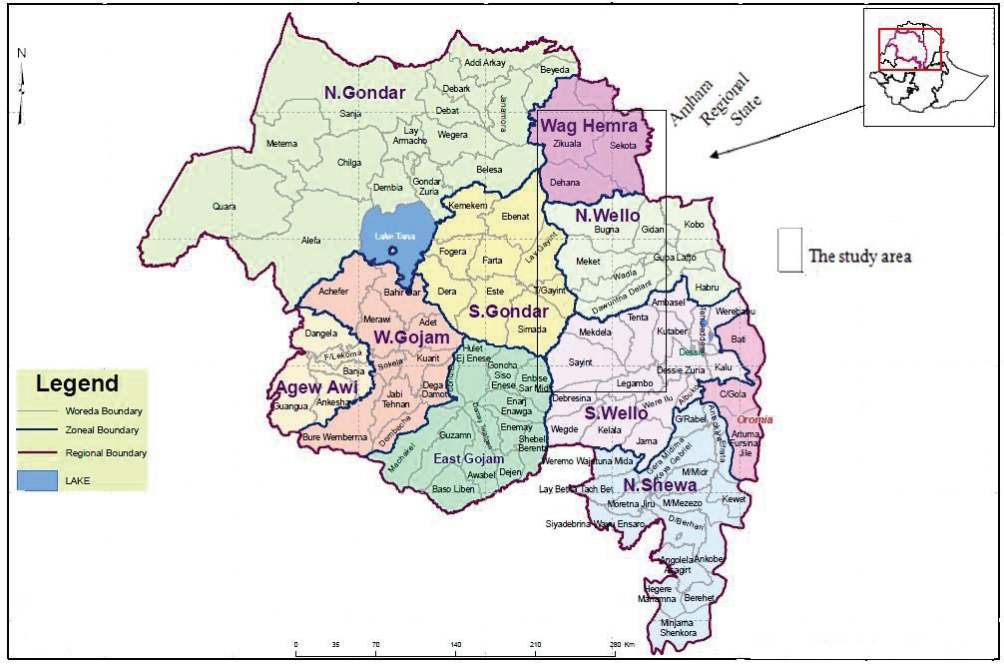
**Map of Ethiopia and the study area**. (source http://www.ocha-eth.org/Maps/downloadables/AMHARA.pdf accessed on 30 January 2010).

The study area is located in the highlands of North Eastern Ethiopia covering three administrative zones (provinces) of the Amhara Regional State: *Wag Hemra*, *North *Wello (*N.Wello*) and *South Wello (S.Wello) *(Figure 1). Eleven barley growing *woredas *(districts) were selected from the three zones: *Sekota *and *Dehana *from *Wag Hemra; Gidan, Gubalafto, Meket *and *Wadla *from *N.Wello; *and *Kutaber, Dessie zuria, Tenta, Legambo *and *Wereilu *from *S.Wello*. *Woredas *are small administrative units within a zone, and a *woreda *is subdivided into smaller administrative units called *kebeles *(peasant associations). The *woredas *are divided into highland (*dega*), intermediate (*woyna dega*) and low land (*kola*) ecologies based on altitude. The study areas covered only highland parts of each *woreda *which fall within 10°50'60N - 12°37'50N latitude and 39°2'5E - 39°10'60E longitude ranges with *Sekota *town in *Wag Hemra *and *Akesta *town in *S.Wello *as the most northerly and southerly places with altitudes ranging from 2000 - 3400 m.

**Figure 2:**
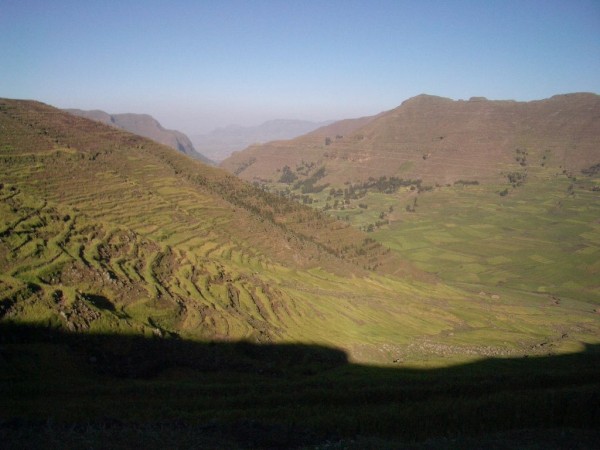
**Showing barley fields in a degraded and steep slope area around *dilb *area in *N.Wello***.

North Eastern Ethiopia is generally characterized by rugged mountains, hills and valley bottoms and all the barley growing places of the *woredas *were accessible by gravel roads. Small land holding (0.5~1 ha) is one of the prominent features of the mixed (crop and livestock) subsistence farming system, and even steep slopes are put into crop production (e.g. Figure 2). Land degradation and low soil fertility are major problems with the situation in *Wag Hemra *being the most affected. Previous studies indicated the need for the application of fertilizers to increase yield (Sirinka Agricultural Research Center, unpublished report). The rainfall distribution is bimodal in *Kutaber, Dessie zuria, Tenta, Legambo*, parts of *Wereilu, Guba lafto, Meket, Wadla, and Gidan*. But in *Sekota *and *Dehana*, parts of *Gidan, Meket, Wadla *and *Wereilu*, the rainfall is uni-modal (Table 1). At times, the rainfall can be erratic in distribution and inadequate in amount, leading to crop failures.

**Table 1:** Mean monthly rainfall for selected stations in the study districts

Zone	District	Station	Months												Total
			**Jan**	**Feb.**	**Mar.**	**Apr.**	**May**	**Jun**	**Jul.**	**Aug.**	**Sep.**	**Oct.**	**Nov.**	**Dec.**	

*Wag Hemra*	*Sekota*	*Sekota*	-	-	-	-	-	-	-	-	-	-	-	-	575.4

*N.Wello*	*Meket*	*Estaysh (6)*	7.0	10.3	79.4	82.0	16.8	20.2	332.7	271.6	46.6	23.1	8.3	7.8	905.7

*N.Wello*	*Gidan*	*Kulmesk (10)*	9.4	0.0	0.0	16.9	0.2	0.0	286.0	285.3	109.7	10.3	29.1	25.6	772.5

*S.Wello*	*Dessie zuria*	*Boru Meda (13)*	33.2	38.8	58.6	102.9	53.0	34.0	386.9	274.9	124.7	66.6	16.7	11.2	1203.5

*S.Wello*	*Dessie*	*Dessie (9)*	39.4	41.3	80.3	102.8	74.4	33.1	326.2	340.7	155.0	73.1	40.6	24.4	1331.2

*S.Wello*	*Kutaber*	*Kutaber (13)*	13.9	16.4	45.5	62.3	67.3	49.5	337.6	323.0	151.3	37.98	16.75	7.8	1129.2

*S.Wello*	*Woreilu*	*Woreilu (7)*	16.2	26.7	46.4	42.6	37.5	33.8	287.8	266.0	54.0	11.3	10.6	5.3	838.2

Source: Sirinka Agricultural Research Center [unpublished data]. *number s in parenthesis next to station names indicate the number of years from which the average was calculated for years 1989 to 2000 for N.&S.Wello. The value for Sekota is long-term average from 1971 to 2004 [source: 50]

### Sampling Procedures

*Kebeles *and peasant households constituted the sampling frame. From each *woreda*, five *kebeles*, and from each *kebele*, five households were randomly selected to provide a sample total of 275 households. The highlands are some of the densely populated areas in the country. In terms of religion, farmers in *S.Wello *are predominantly Muslims and those in *N.Wello *and *Wag Hemra *zones are predominantly Christians. Ethnicity wise, farmers in *N.Wello *and *S.Wello *are *Amhara*, and those in *Wag Hemra *include *Amhara *and *Agew *(Table 2). *Amhara *is one of the largest ethnic groups accounting more than 25% of Ethiopian population [17,18]. Except *Sekota*, more than 99% of the dwellers in the study districts are Amharic speaking people.

**Table 2:** Area, altitude and population demography of the study districts

District	Area (sq. km)	Altitude (masl)**	Population	Ethnicity	Main Language spoken	Religion*
***Wag Hemra***						

*Sekota *	1,722.43	1100 to ≥ 3810	112,396	Agaw/Kamyr (74.24%) Amhara (22.57%) Tigrayan (3.06%)	Amharic Kamyr	Christian (99.34%)

*Dehana *	1,643.07	-	109,725	Amhara (98.74%) Agaw/Kamyr (1.11%)	Amharic	Christian (99.8%)

***N. Wello***						

*Gidan *	1,089.80	1300 to ≥ 4100	158,428	Amhara (99.96%)	Amharic	Christian (99.63%)

*Gubalafto *	900.49	1300 to ≥ 3900	139,825	Amhara (99.92%)	Amharic	Christian (88.55%) Muslim (11.42%)

*Meket*	1,909.25	1200 to ≥ 3000	226,644	Amhara (99.95%)	Amharic	Christian (94.69%)

*Wadla *	855.29	700 to ≥ 3200	128,170	Amhara (99.94%)	Amharic	Christian (96.21%)

***S. Wello***						

*Kutaber *	719.92	800 to ≥ 3200	95,410	Amhara (99.86%)	Amharic	Muslim (88.65%) Christian (10.78%)

*Dessie zuria*	937.32	1800 to ≥ 3500	157,679	Amhara (99.93%)	Amharic	Muslim (97.72%) Christian (2.21%)

*Woreilu*	740.96	1700 to ≥ 3200	109,244	Amhara (99.91%)	Amharic	Muslim (80.04%) Christian (19.83%)

*Legambo *	1,017.35	1500 to ≥ 3700	165,026	Amhara (99.9%)	Amharic	Muslim (92.99%) Christian (6.82%)

*Tenta *	1,316.34	600 to ≥ 3700	166,239	Amhara (99.93%)	Amharic	Muslim (77.92%) Christian (21.95%)

*Source: *CSA (1994, 2000, 2007, http://en.wikipedia.org/wiki/List_of_woredas_in_the_Amhara_Region accessed on 20 January 2011);*Orthodox Christianity and Suni Muslim;**In Ethiopia, agro-ecologies are traditionally categorized into low land (<1500 m), intermediate (1500 - 2000 m) and highland (>2000 m) above sea level. Our study covered only the highland areas.

We visited individual farmers' places (home or farm) to gather the information. Using an open ended questionnaire, interviews and discussions were conducted involving the men and women at times to get information on practices that require specific knowledge and skills of either member of the household. Women are traditionally responsible for preparing foods, and therefore, more knowledgeable about food preparatory techniques and cooking qualities. The farmers were asked to describe the cropping practices, the types, names, characters and quality attributes of landraces grown, the types of barley foods and beverage products and their preparations. The information collected was more descriptive of the practices rather than quantitative measurements. In some cases, the discussions were turned into group discussions with the neighbours turning up for curiosity. Their involvements in the discussions were entertained, and consensus opinions were taken. Development agents, subject matter specialists of agricultural office of the districts and administrative staffs of *kebeles *cooperated in contacting farmers for the discussion. Information from all the study areas was summarized, and where differences were observed from areas to areas, such cases were indicated. Whenever possible, secondary data were surveyed from published sources.

## Results

### Importance of barley

According to the farmers, barley is the king of crops ("*gebs ye ehil nigus"*) and it is preferred to other crops. Some of the reasons for this as stated by the farmers are summarized (Table 3). It is the number one crop both in terms of acreage and production in the surveyed areas produced during both *meher *(main rain) and *belg *(small rain) seasons. In the higher altitude areas (> 3000 m), barley is the only crop with linseed and potato cultivated in few areas and on very small plots. Wheat, faba bean, field pea, linseed, lentil, maize, potato and tef are important components of the cropping system in some areas, particularly in areas with altitudes from 2000 - 2400 m. For example, farmers in *Dessie Zuria *and *Kutaber *grow maize, pulses and tef during the *meher *season on plots that are relatively well drained and not affected by frost, and grow barley during *belg*. Similarly, in *N.Wello *and *Wag Hemra*, barley is grown along with wheat, tef and various pulses. Barley is less important in areas below 2000 m. Over all, barley ranks 3^rd ^or 4^th ^in terms of area and production in the three zones (Table 4).

**Table 3:** Reasons why farmers prefer barley and their importance

Reasons	Importance
• Suitable for high altitude, performs better than other crops	Very important
• Can be produced both in *belg *and *meher *seasons	Very important
• Tolerant to weather and agronomic stresses like frost, water logging, weeds, diseases, and insects	Very important
• Suitable for many kinds of dishes (including *injera) *with a better taste	Very important
• Good source of energy and consuming barley foods gives body strength	Very important
• Medicinal purposes for gastritis, headache and can heal broken bones and fractures	Very important
• The best choice for local beverages	Very important
• Relatively high yielding with low management	Important
• Produces high quantity and quality straw for feed, which is preferred by animals	Important
• Quality straws for roofing (thatching) houses and bedding	Important
• Grain, flour and food products store better than other crops	Important
• Good cash crop as it is highly demanded for local beverages prepared in towns for sale	Less important

**Table 4:** Area coverage, production and rank of barley in the three administrative zones

Administrative levels	area (,000 hectare)	%	production (,000 quintal)	%	rank	number of administrative units*
*Wag Hemra*	12.98	14.08	94.12	16.65	3^rd^	3 districts

*N.Wello*	33.95	14.61	292.34	12.87	3^rd^	8 districts

*S.Wello*	25.1	7.01	239.37	6.79	4^th^	15 districts

*Amhara Regional State*	287.87	8,3	2488.52	7.53	5^th^	10 zones

*Ethiopia *	874.0	9.25	9454.2	8.91	5^th^	9 Regional States

Source: CSA (2000); *includes districts, zones and regional states where barley may not be important. Barley is the third important crop in North Wello and Wag Hemra after tef and sorghum while it ranks 4^th ^in South Wello after tef, sorghum and wheat. At the regional state and national levels, barley ranks 5^th ^after tef, sorghum, maize and wheat. Oromiya, Amhara and Tigray Regional States account about 87% of barley production in the country.

Farmers store barley grains and seeds in a well-prepared underground pit to protect from weevils and molds damage as well as other physiological changes that cause loss of viability. Produces from different landraces are stored separately unless they are grown in mixtures. From interviews, barley grains can be stored for 5-25 years depending on the storage conditions, with dry and cold places being ideal for long storage. However, nowadays farmers hardly produce any surplus that can be stored for more than a year. Very small amount of barley grain is sold to generate cash.

**Figure 3:**
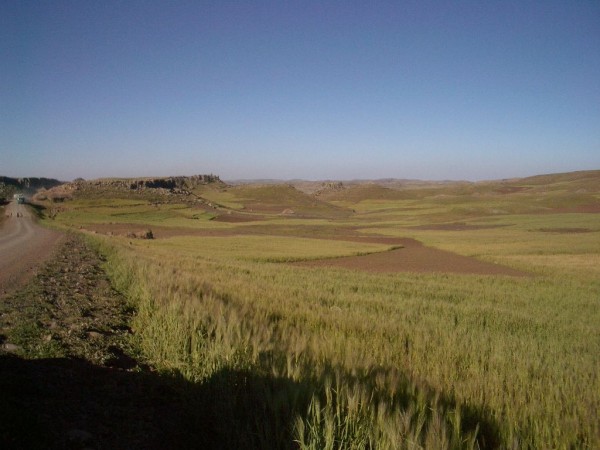
***Meher *season barley fields in October around *Kebero meda *locality *(N.Wello) *side by side with plots prepared for belg planting**.

*Meher *production is the predominant system in *N.Wello *and *Wag Hemra *while *belg *is the predominant system in *S.Wello*. The *meher *season is through May to December (with July and August being the main rainfall months), while the *belg *season is through January to July, with mid January to end of February/early March considered as the best *belg *planting time for late type landraces to harvest before the *meher *season rain starts. Otherwise, early maturing types can be planted as late as April and harvesting may go into July where there is a risk of damage by the main season rainfall. *Belg *barley producing areas, mainly *S.Wello*, are characterized by black soils which are prone to water logging. Consequently, flat plains or low laying areas are not workable during the main rain season. If planted on such plains, the performance of barley would be very poor. There is also a frost problem in September/October. Therefore, majority of the fields in these areas are left flooded during the main rainy season and land preparation is through September to December. This practice is noticeable in *Gragn meda *and *Guguftu *(*Dessie zuria*) and *Gimba *(*Legambo*), where fields are left fallow during the *meher *season, and covered with barley crop during the *belg *season. The implications of insufficient *belg *rainfall and subsequent crop failures are serious on food security of these areas. The situation is, however, different in *N.Wello*, where it is common, during the *meher *season, to see barley crop fields side by side with fallow fields left for *belg *barley planting (Figure 3). The *belg *season is less dependable except some areas with water logging and frost problem where farmers rely mainly on the *belg *barley production. In general, if farmers fail to plant during the *belg *season, they may still be able to plant barley during the *meher *season. This is not to imply that the *belg *barley system is not important in *N.Wello *but to indicate that *belg *crop failures will have more localized livelihood impacts compared to *S.Wello*.

### Ethnobotany and Types of landraces grown

Farmers in the study districts purposely maintain landraces to address various needs. These needs included, but not limited to, suitability for early or late planting (i.e. maturity), yield potential in relation to the type of environment intended to be grown, conditions of the soils (i.e. water-logged, fertility or frost effects), and intended dishes and beverages (includes quantitative and qualitative aspects such as product volume, taste, visual appeal, color, storability etc). Fifteen landraces were grown, which vary in maturity, yield potential, stress tolerance, end-use qualities and other agronomic traits. Table 5 highlights the ethno-botany of some of the landraces grown by farmers, which gives some idea about the richness of landraces types and their management as described by the farmers. The naming and descriptions of the landraces reflect key quantitative, qualitative traits and end-use qualities as well as other information such as planting time or origins. The most common landraces listed by farmers were *Nechita, Ginbote, Wongada, Sene gebs, Ehil zer, Enat gebs, Wogere, Meher gebs (Ere), Ahya Asin, Tikur gebs, Gendit, Agere, Tsebel, Zibna *and *Temej*. Majority of these landraces are six row types. The landraces from *Wag Hemra *are early maturing and two rowed that are adaptable to low moisture and short growing *meher *season of the area.

**Table 5:** Vernacular names & their meaning as well as descriptions of major barley landraces grown in North Eastern Ethiopia

Names	Zones	Meaning	No of rows*	Seed colour	Maturity	Preferred use**
*Agere*	*N*. and *S.Wello*	The landrace has been cultivated in the area for long time and it is not considered as introduction from somewhere else.	six	white	Medium	*Injera*

*Ahya Asin*	*N.Wello*	*Asin *refers to the heaviness of the grain for donkey *(Ahya) *to carry	six		*Medium *	*Injera *

*Ehil zer*	*N.Wello*	*Ehil *and *zer *mean crop and seed, respectively. The context of the naming implies the earliness of the landrace to produce some seed irrespective of the growing condition (moisture stress, poor soil, frost)	irregular	purplish	Early	*Beverages *

*Enat gebs*	*N*. and *S.Wello*	Barley as good as mother *(Enat)*, and best of all the landraces.	six	white	Late	*Injera, beverages*

*Gendit *	*N.Wello*	The landrace has a very long spike (head).	six	white	Medium	*Injera, beverages *

*Ginbote*	*N.Wello*	A landrace planted in May *(Ginbot)*.	six	white	Late	*Injera, genfo, kolo, kinche *

*Meher gebs (Ere)*	*N*. and *S.Wello*	Barley landrace grown during the *meher *season.	six	white	Medium	*Injera *

*Nechita*	*N.Wello*	The name refers to the whiteness of the seed. It has whiter seed than other landraces.	six	Bright white	Medium	*Genfo, kinche, kolo*

*Sene gebs*	*N*. and *S.Wello*	Barley planted in June *(Sene).*	irregular	white	Medium	*Injera*

*Temej *	*N*. and *S.Wello*	Hull-less barley	six	white	Medium	*Kolo *

*Tikur gebs*	*N*. and *S.Wello*	black (*Tikur*) color of the barley grain	six, irregular	Black	Medium	*Beverages *

*Tsebel *	*Wag Hemra*	Barley landrace that produces grain with very low rainfall, a rainfall as small as holy water (*tsebel*).	two	white	Early	*Injera, beverages *

*Wogere *	*N.Wello*	-	six	white	Medium	*Injera, beverages *

*Wongada*	*N.Wello*	-	six	white	Medium	*Injera *

*Zibna*	*Wag Hemra*	-	two	white	Early	*Injera, beverages *

* If the soil is fertile, irregular row type landraces like Ehil zer grow to have six rows;**preference depends on ability to grow the landrace per se. There is no landrace, except Temej, that cannot be used for *injera *which is the main dish of the study area. In other words, the less preferred ones are used for making *injera *if preferred ones are not available. For example, *Ehil zer *is widely used for making *injera *in *N. Wello *because it is the landrace that is relatively better adapted and stable yielding compared to other landraces (e.g. *Enat gebs & Ginbote*).

All the landraces listed by farmers are hulled except *Temej*. The landraces may be planted as pure stand (the dominant system) or in mixtures. If planted in mixtures, usually one or two landraces dominate the mixture. Although frequently mentioned as an important landrace, *Tikur gebs *was observed grown in mixture with other landraces with hardly any pure stand indicating the preference for white seeded types which cover wider areas. The farmers also mix-plant barley, particularly in *N.Wello*, with wheat, and this practice is known as *Wasera*. The number of landraces mentioned was higher in *N.Wello *and *S.Wello*. In areas where both the *belg *and *meher *seasons plantings are practiced, majority of the landraces may be planted either in the *belg *or *meher *season depending on the onset of the rainfall. For example, the *belg *rain occasionally starts very early making it possible to plant late maturing types including the ones usually grown during the *meher *season. Landraces like *Ehil zer *are grown both in the *belg *and *meher *seasons. In fact, farmers may use the produce from *meher *as a seed source for *belg *planting, and vice versa.

### Barley Foods and their Preparation

**Figure 4:**
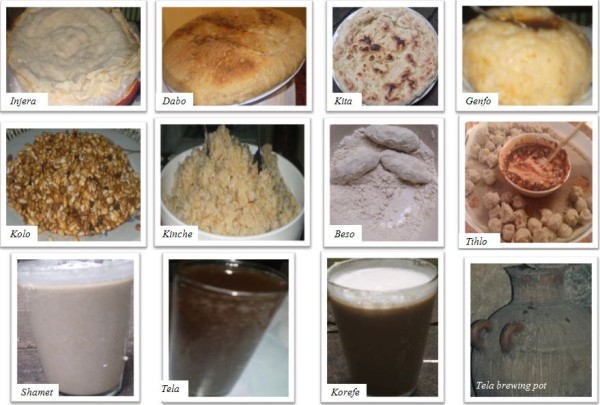
**Some dishes and local beverages prepared from barley**. (The source for *Tihlo *picture is: http://nutritionfortheworld.wetpaint.com/page/Tihlo accessed on 20 April 2011).

More than 20 types of traditional barley dishes and beverages were reportedly prepared from barley. The food and beverage products are prepared from ground/milled barley flour, whole/cracked grains, roasted or boiled grains for main, side, ceremonial and recuperating dishes. Some of the dishes and beverages prepared from barley are shown (Figure 4). The food value of barley as sources of energy is highly acknowledged by the farmers. Some dishes are served to breast-feeding mothers with the belief that they enhance breast milk production. Besides, some dishes are claimed to be a remedy for gastritis, while some others are reported to be a good substitute for breast milk; good to heal broken bones and fractures. For foods prepared from flour, the milling of barley is done either by special stone mill (traditional hand-grind grains using a stone grinder) or motorised mill. The flour can be stored from 6 months to 10 years depending on the temperature of the area with high temperature storage places increasing the rate of deterioration. Containers made of clay (pots) or mud and/or animal skin (*akimada*) are used for storing flour. Some farmers in *S.Wello *reported that flours from traditional stone mills store better than from motorized mills. This could be related to more frictional heat with motorized mills leading to hotter milled flours. Nishita and Bean [19] have measured temperatures up to 75°C during milling of rice. Motorized milled are also expected to grind finer than manual mills, and the increase in surface area from finer particles possibly exposes barley components more to deterioration. Table 6 summarizes the different dishes and beverages prepared from barley. A more detailed description of the different dishes and traditional beverages prepared by the farmers of the study area is presented below along with their preparations.

**Table 6:** A brief description of the types and preparation methods of barley dishes and beverages in North Eastern Ethiopia

Name	Method of preparation/processing	Frequency of use
*Injera *	A leaven bread made from raw grain flour with the dough fermented for 2-4 days and baked on clay pan	• It is the main daily dish

*Kita*	Instant bread baked from unfermented dough of raw grain flour	• Occasionally when no *Injera *or *kolo *is available.
		• Usually prepared for children

*Dabo *	Thick bread baked from fermented dough of raw grain flour	• Not common
		• Wheat is preferred

*Kolo*	Roasted grain prepared from dehulled barley	• Prepared daily as additional food to the main meal
		• Also used as travelling food

*Nifro*	Boiled grain prepared from dehulled barley	• Prepared occasionally
		• Mostly prepared for cultural occasions

*Beso *	Solid food prepared from roasted barley flour and water	• Prepared occasionally
		• Relieves gastritis
		• Used as travelling food

*Shamet *	Semi fluid drink made from roasted barley flour	• Prepared occasionally by farmers
		• Daily for sale in towns
		• Relieves gastritis

*Genfo *	Thick porridge prepared from raw or lightly roasted grain flour	• Commonly as a substitute or when other dishes are not prepared
		• Commonly prepared for postnatal mothers

*Kinche *	A dish prepared from cracked raw barley grains. Somewhat equivalent to oat meal.	• Occasionally as a substitute to other dishes
		• Breakfast

*Muk *	Gruel made from raw barley grain fine flour	• Occasionally for a change
		• Mostly prepared for children and sick person

*Shorba *	Semi fluid drink prepared from cracked grain	Occasionally and commonly during Ramadan

*Tihlo *	Prepared from roasted barley flour and water, served with sauce	• Occasionally and it is only known in *Wag Hemra*

*Tela*	Alcoholic beverage prepared from *gesho*, malt, roasted grain (*derekot or asharo*) and *kita*	• Prepared mainly during holidays/traditional ceremonies
		• Commonly prepared and sold in towns

*Bukre*	Non-alcoholic beverage prepared from malt, roasted grain and *kita*	• Prepared occasionally, usually for holidays
		• Prepared by Muslims

*Korefe*	Alcoholic beverage prepared from *gesho*, malt and lightly roasted barley grain and *kita*	• Occasionally for holidays
		• Usually prepared and sold in towns

*Filtered tela*	An alcoholic beverage similar to *tela *with a slightly different preparation and higher alcoholic content	• Occasionally during holidays
		• Sold in towns

*Eshet*	Barley seeds consumed green at milky/dough stage	• Consumed in the field during grain filling stage mainly by children minding livestock.

*Enkuto *	Flame roasted mature dry spikes consumed in the field	• Occasionally during harvest time

*Areke *	Alcoholic spirit	• Occasionally and wheat is preferred

*Tea/coffee*	Drink from dark roasted barley	• In very limited cases and when proper tea/coffee has run out.

#### i) Barley Foods from raw-grain flour

The main food products prepared from raw barley grain flour are *injera, kita and dabo. Injera*, an unleavened thin pan cake, is the main dish and daily diet of the people in the area served with sauces. *Enat gebs, Sene gebs *and *Meher gebs *are the preferred landraces . The grain is well dried, cleaned, dehulled using mortar and pestle, heated lightly and milled in to moderate fine flour, sieved and dough is prepared. The dough preparation and other procedures for making *injera *are well-documented [20], and they essentially tally with the reports from the farmers in the study area with some variations. For example, fermentation can be for 2-4 days, but, if time is limited, the dough can be fermented for only one or two days. The *injera *from well fermented dough makes a better sourer taste and has good storability. The higher is the altitude, the longer is the fermentation time required as temperature would be lower. Lactic acid bacteria and yeasts are the main fermentation organisms in *injera *[20], and their products coupled with a drop in pH would stabilize *injera *during storage. Besides, in some areas, particularly in *S.Wello*, small quantities of malt flour as well as *erimito *are added during dough making to improve the *injera *quality and storability. *Erimito *is prepared from coarsely ground barley flour by mixing with water to form thick dough, which is made into small balls and stored for couple of weeks. A good *injera *is soft, fluffy and spongy with good and well distributed 'eyes' and it does not break when rolled. Farmers stressed that the skills (*moya*) of women in getting the right balance in the fermentation and baking process affect the quality and storability of *injera*. Women usually put leaves, plastic or pulses grains on the *mesob *(*injera *storage made of woven grass) under the *injera *to improve its shelf-life . The *injera *is baked on clay pan, *mitad *(also known as *mogogo *in some areas). Wheat, tef, sorghum, and maize flour can be mixed with barley flour for making *injera*.

For making *kita*, the flour is mixed with water and kneaded by hand with a pinch of salt to make thick unfermented dough. Then, it is baked immediately on both sides using a clay pan (*mitad*) or iron pan (*biret mitad*) by turning after being baked on one side. *Kita *is a relatively thicker and harder bread but smaller in size (about the size and thickness of a pizza base) compared to *injera*. It is served either with butter, milk, or linseed paste. It is instant bread usually prepared for immediate consumption for children or as an emergency food when no *injera *or *kolo *is available. The term *kita *is used for any product prepared from unfermented dough with or without qualifying it. *Dabo *is leavened homemade bread, which is much thicker and softer than *kita*. The dough is prepared thick with salt added for an overnight fermentation. A leaven (*ersho*) is added as a starter of fermentation, which is also the case for *injera*. The *ersho *is usually obtained by saving a small amount of the previous *injera *dough. *Dabo *is baked on both sides by burning fire on both sides after covering the top with leaves/mud/clay. It is usually prepared for holidays or cultural gatherings. Although wheat is the preferred crop for *dabo*, barley is used where it is the only option in higher altitude areas. The flour for *kita *and *dabo *is taken from that prepared for *injera*.

#### ii) Barley foods from roasted/boiled whole grain

Various roasted and boiled barley foods are known to the study area, but *kolo*, a roasted grain, is the most widely consumed. To prepare *kolo*, the grain is dehulled using hot water directly or after soaking in water for few hours to facilitate dehulling. The dehulling is carried out mechanically by pounding the hot water treated or soaked grains using mortar and pestle. The grains are heated on the sun or on iron/clay pans to dry the hulls, which are subsequently blown prior to roasting. Then the grains are roasted and lightly pounded using mortar and pestle or hand rubbed to remove the remaining hulls followed by final blowing. *Kolo *is consumed sole or mixed with roasted field pea, faba bean, safflower or chickpea. It is usually consumed as a snack dish served before the main dish, and during coffee ceremony and other cultural occasions. *Kolo *is also a good travelling food as it stores well. *Temej, Nechita and Ginbote *are the preferred landraces for *kolo.*

The same dehulled whole grain barley for *kolo *can be boiled sole or mixed with pulse to make *nifro *to be served as a snack. Barley *nifro *is not as common as *kolo *with other crops such as wheat, and pulses being preferred. It is mainly prepared for cultural occasions. Another roasted whole grain food types are *eshet *and *enkuto*, which are consumed in the field before the crop is harvested. Barley spikes at dough stage (*eshet*) are consumed as raw green grains or flame roasted by children , or dry (matured) spikes are flame roasted (*enkuto*) and consumed . Another form of roasted barley uses includes barley tea and coffee. Whole grain barley is roasted dark colour and boiled to make barley tea, or mixed with roasted coffee and ground using mortar and pestle to make coffee. But, the use of barley as tea and coffee is very limited in the area.

#### iii) Barley foods from roasted-grain flour

At least five types of food products are prepared from roasted barley grain flour:*genfo, muk, beso, shamia *and *tihlo *with *genfo *(porridge) and *beso *being more commonly consumed. Barley grain for *genfo *is partially dehulled using mortar and pestle, and sun-dried or most often lightly roasted to, according to the farmers, increase "water uptake ("*wuha endiyanesa*") during cooking so that high volume *genfo *can be obtained from a small amount of flour". The high volume could be as a result of partial gelatinization of starch in the grain due to roasting. Gelatinized starch generally absorbs more water, and swells more than non-gelatinised starch [21]. The lightly roasted grain is milled and sieved to remove remaining hull. The flour is added with some salt in boiled water and cooked with occasional stirring. *Genfo *is served hot either in the pot or in a bowl with spiced butter, honey, *berbere *(spiced paprika/chilli) or linseed paste. The dish is usually prepared for post-natal women or for a sick family member. But, it is also prepared sometimes as a variety dish, as a substitute for a common dish as it takes less time to prepare. In general, *genfo *is preferred as a breakfast food.

The grains for *beso *may or may not be dehulled before milling. It is a common practice to roast the grains first and partially dehull with mortar and pestle before coarse milling and sieving followed by fine milling and subsequent sieving. *Beso *is prepared using cold or hot water to moisten the flour on a bowl in such a way that it can be balled/rolled using hand and served. Salt is usually added in the water, but sugar or melted spiced butter can also be added if available. According to the farmers, *beso *cures gastritis. It also helps to alleviate food shortages during September - November because the matured grain, which is not very well dried yet and cannot be used for other dishes, can be harvested from field and threshed to be used for preparing *beso*. *Tihlo *is prepared in *Wag Hemra *zone. The processing of barley for *tihlo *is similar to *beso *but the grain is completely dehulled and the milling requires extra care to avoid mixing with flours from other crops which might decrease the quality. Also, more water is used to prepare *tihlo *than *beso*. *Tihlo *is usually balled by hand and served with freshly made hot *shiro wot *(sauce made from pulses flour and spices).

The flour for *shamia (shamet) *is the same with *beso *flour, but *shamia *is prepared as a drink. *Beso *flour is mixed with cold water plus sugar, and served in a cup or glass. *Shamia *is not prepared frequently, and it is considered as a luxurious food item as sugar might not be readily available. Farmers prepare *shamia *mostly when someone suffers from gastritis as it is considered medicinal. It is more commonly prepared in towns for sale. *Beso *flour is a preferred travelling food ("*yemenged sink"*) as the dishes are easy to prepare, and the flour can be stored for long with no quality deterioration. *Muk (gruel) *is a very smooth semi-solid drink. The barley grain is dehulled and milled into fine flour and sieved. The flour is added to boiling water and cooked with occasional stirring. Once cooked, it is usually served hot with sugar. Muk is usually prepared for sick people and children, but it is also a favorite drink by women.

#### iv) Barley foods from cracked grain

*Kinche *and *shorba *are the two most important dishes prepared from cracked barley grains with *Nechita *and *Ginbote *as the preferred landraces. For *kinche*, the grain is dehulled using mortar and pestle, roasted very lightly, cracked into four or five parts, sieved and cooked in boiled water with occasional stirring to get a thick consistency. Sugar is added and *kinche *is served when it is cold, and spiced butter, if available, can also be added. It is considered as a luxury food and, therefore, prepared occasionally for changing diet and/or as an alternative dish when other dishes are not readily available. The preparation of barley for *shorba *(*soup) *is the same to that of *kinche *except that more water is added to *shorba*. Thus, it is a drink served hot in a cup or using spoon in a bowl. It can be mixed with some vegetables and pulses but it is usually served sole with sugar, salt and spiced butter (if available). It is a very important dish during *Ramadan*, when it might be prepared daily.

#### v) Traditional Beverages

*Tela, filtered tela, korefe, bukre *and *areke *are the various beverages locally prepared from barley *. Tela *(also known as *zilil *in some *woredas*) is the most common and preferred local beverage. It is usually prepared for annual and religious holidays, and traditional ceremonies, but also for sale in towns and cities. The ingredients for making *tela *are barley malt, *gesho *(*Rhamnus prinoides*), *derekot *(or *asharo), kita *(qualified as *tela kita*) and water. The brewing clay pot (*gan*) is washed several times and smoked with locally available selected shrubs to properly clean it. Barley malt flour and dried *gesho *leaves (ground by mortar and pestle using water), are mixed with water in the *gan *and left for 2-5 days to ferment and yield. This is called *tinsis*. The purpose of *gesho *seems to be similar to hops in commercial brewery as it has a bitter taste and adds a bitter flavor to *tela *by balancing the sweetness of the malt. The *kita *is prepared by lightly roasting barley and milling before a non-fermented dough is prepared and baked. The *kita *and pounded *gesho *stems are added to the *tinsis *and allowed to ferment overnight. Simultaneously, another barley grains are boiled, dried and roasted black to make *derekot*, which is then milled to flour and added, in equal amounts to *kita*, to make *difdif*. When the *difdif *is fermented well for 3-4 days, enough water is added and the pot is sealed to make *tela*, which is usually left for 5-7 days to make purified and clarified quality *tela*. When the clarified *tela *is used, fresh water could be added and left overnight to ferment to get secondary and weaker *tela *called *kirare*. The leftover (byproduct) after the *kirare*, called *atela*, is commonly fed to animals. The *derekot *can be substituted by *asharo*, which is prepared simply by roasting black the barley grain without boiling. *Tela *from *derekot *is preferred. But since it is tedious and takes long time to prepare the *derekot *(boiling, drying, roasting etc), its use is limited, and *asharo *is commonly used to prepare *tela. Derekot *is usually used when a big cultural ceremony is planned like wedding where the hosts would brew a quality *tela*.

In *N.Wello*, women keep part of the *difdif *(made from *derekot*, not *asharo) *in a separate clay container for up to three or more months, from which quantities are taken to prepare *tela*. The name, *zilil*, for the *tela *implies the practice of saving part of the *difdif*. A small quantity of the *difdif *is taken to a brewing pot and more water added to make the *tela *that would be ready to use after overnight fermentation. *Filtered tela *is made in the same way as *tela*, but more concentrated with higher alcohol content due to less water added, and filtered through a cotton cloth (this makes it clearer than *tela*) and kept in a sealed container. It can be kept for a longer period of time, upto three weeks. *Korefe*, another form of *tela*, is prepared mainly in *N.Wello *and *Wag Hemra *zones. The preparation is similar to *zilil *in that the *difdif *is kept for a long time, and a part is used to make *tela *that ferments overnight. But the *difdif *is filtered in a pot using clean cotton cloth by adding water similar to *filtered tela*. If it is filtered in the afternoon, it would be ready to serve in the morning of the next day. While the other forms of *tela *are brown, *korefe *is lighter in color because the *asharo *is not roasted very dark. Normally, *korefe *is not purified and clear when served and it has white bubbles (*korefe*).

*Bukre *is another beverage mainly prepared by Muslims, and its preparation is similar to *tela *except that *gesho *is not added. *Bukre *is considered as alcohol free drink although the preparation follows a lot of fermentation process. *Areke *is a distilled spirit with preparation and fermentation process similar to *tela*. Instead of adding more water to make clear *tela*, the fermented product (*difdif*) is boiled in a sealed clay pot and the steam is distilled. Barley is not the first choice as other crops are preferred for quality *areke*.

## Discussion

### Importance of barley

For the subsistence farmers of North Eastern Ethiopia highlands, barley is the crop of choice for many reasons (Table 3). Although some of these reasons have been proven scientifically (e.g. tolerance to frost and storage stability) [22,23], many other reasons could initiate scientific investigations. Some of the areas are above tree border line (adaptation zone) where no trees or shrubs are observed (e.g. Figure 3). Only barley can be planted in these areas. By the farmers' estimates, about 90% of the produce is used for home consumption, of which about 10% is for local beverages while the rest is for food. This figure is higher than 40% [7] and 79% [24] quoted earlier for national consumption of barley-based foods in Ethiopia. This could be attributed to the subsistence nature of the farming and also to the study area being drought prone limiting food security of farmers. Out of the 105 *woredas *in the Amhara Regional State, 48 *woredas *are drought-prone and food-deficit [25]. All the *woredas *in our study fall in this category.

Farmers grow many landraces, which vary in maturity, yield potential, stress tolerance, end-use quality, and other agronomic traits. The landraces are purposely maintained to address various needs. Similar reasons were noted why farmers kept many landraces of barley in central Ethiopia [15], potato in the Andes [26], rice in China [27], various crops in Italy [28], sorghum in *S.Wello*, Ethiopia [29], maize in Mexico [30], and durum wheat in East Shewa, Ethiopia [13]. The different landraces planted in the study areas were identified, named and described by the farmers. Studies on other crops showed that vernacular names and farmers' descriptions of landraces can relate to formal scientific classifications. For example, in *S.Wello*, Teshome *et al*. [6] found that the sorghum landraces named by farmers could be discriminated very well as distinct types on the basis of formal taxonomic classification using morphological characters. We did not attempt to characterize the phenotypic (quantitative or qualitative) traits of each landrace grown by the farmers.

### Diverse use of barley for dishes and beverages

According to the farmers, there is no other crop they know or are aware of that is as suitable as barley to prepare many of the dishes and beverages known in the study areas. The expression "barley is the king of crops ("*gebs ye ehil nigus"*)" emphasizes its suitability for diverse use. More than 20 types of traditional barley foods and beverages were described by farmers. Some of these dishes and beverages like *beso*, *shamia*, *tihlo*, *korefe *and *genfo *are exclusively prepared from barley. Some dishes and beverages may be prepared from other crops. For example, *tela *and *bukre *may be prepared from sorghum or maize, and *kolo *can be prepared from wheat or pulses, but the quality and taste would not be as good as that from barley. As it was reported for farmers in central Ethiopia [15], the various barley foods and drinks also play an important role in the socio-economic and cultural life of farmers and urban dwellers of the study area. Special events like wedding, annual festivals and ceremonies are celebrated with foods and drinks of barley. Traditionally, it is a custom in many parts of Ethiopia (both rural and urban areas) to prepare barley flour for *genfo *for an expectant mother and barley is the crop of choice. A postnatal mother eats *genfo *with spiced butter for breakfast and her guests are also served *genfo*. Neighbours and close relatives usually prepare barley *genfo *and take to the new mother. Besides, *tela *is sold in small towns and cities as a source of income for many families.

Although, there could be differences in the method of preparation/processing, preference for certain crop types (e.g. tef, wheat, maize or barley), types of crop varieties (landraces, improved) and naming of dishes (due to language differences), many of the dishes and beverages reported to be prepared in the study areas are also widely prepared in other parts of the country. For example, *injera *is the stable diet of majority Ethiopians; *tela *and *kolo *are also widely consumed. Studies on barley in central Ethiopia [14,15] listed many of the dishes and beverages described by farmers in the study area. However, some of the dishes and beverages like *korefe, zilil *and *tihlo *seem to be specific to this part of the country. On the other hand, *borde *(a beverage), *kori *(a roasted grain coated with spiced butter) and *chiko *(a solid food made from *beso *flour and ghee) described by [15] were not familiar in the study area. Besides, the barley landraces cultivated in central Ethiopia [15] were entirely different (at least in name if not genetically) from the ones in our study areas. Generally, the skills and methods of preparation/processing of the different dishes and beverages are likely to be affected, among others, by the types of crops grown in a particular area. In the lower and intermediate areas where barley is not the dominant crop, *injera *is mainly prepared either from tef, sorghum, maize or wheat. In towns and cities, tef is usually the preferred crop for making *injera*. *Injera *from sorghum or maize would probably have a relatively different processing method. Even for the same crop, adding *erimito *and malt in the dough making process for *injera *from barley seem unique practices to the study area. But the overall approach of preparing *injera *such as the need for fermentation, use of starter and baking essentially remain similar across the country [15,20].

### Health benefits of barley dishes

In addition to the food values, the farmers often emphasised the medicinal properties and health benefits of the different dishes prepared from barley. For example, *shamia *is believed to be a remedy for gastritis; *muk *can serve as a substitute for mother's milk for children. *Muk, genfo, kinche *and other barley foods are believed to heal broken bones and damaged body parts. Besides, these dishes are considered smooth and easily digestible to serve to sick person who cannot take another form of food for quick recovery. The tradition of preparing *genfo *for postnatal mothers is related to the benefit of barley for quick recovery from the effect of child birth. The farmers also mentioned that barley is often fed to broken or rested ox, a valuable asset for ploughing the land, for quick recovery. Some of the health benefit claims about barley foods by the farmers are believed to have scientific basis, which is well documented [31-37]. Barley contains β-glucan soluble fibre and antioxidants, vitamins, minerals, and phytonutrients such as phenolics and lignans, which can reduce the risk of coronary heart disease, cholesterol absorption, diabetes and certain cancers. Such health benefits have created a renewed interest in barley for food. For example, the USA Food and Drug Administration has issued a health benefit endorsement for barley based on β-glucan effects on lowering blood cholesterol and reducing the risk of heart disease [38].

The development of a wide range of barley types allows for targeting barley cultivars to specific end uses [7]. There has been a focus on the development of improved hull-less barley with low or zero amylose (waxy), and high amylose content. Waxy cultivars typically have higher viscous soluble fibre, β-glucan levels than non-waxy types. Hull-less cultivars also permit greater ease in milling and pearling with enhanced processing yields [32,39-41]. The hull-less type of Ethiopian barley constitutes the genetic pool from which the lysine-high protein, hiproly barley was recovered by screening [7,42]. Throughout Ethiopia, the frequency of hull-less barley is low [7]. In North Eastern Ethiopia, hull-less barley is only used for *kolo*, and it is not preferred for other dishes. Besides, it is low yielding and it is only grown on small plots around homestead or mixed with other landraces.

### Relationship between end-use qualities and diversity of landraces

Crop selection and maintenance decisions are generally made based on a set of criteria that has resulted in a complex and continually evolving collection of landraces. The selection criteria often reflect adaptations to changing farming conditions, and responses to the socio-economic and cultural factors that shape farmers priorities [15,26-30]. Diversity in end-uses is one of the important factors that influence the maintenance and genetic diversity of a particular crop. As highlighted in the previous sections, barley is put for diversity of uses in North Eastern Ethiopia, and the livelihood of highland farmers depends on barley. Not all landraces are equally suitable to make the various barley dishes and beverages prepared in the area. Different landraces are preferred for specific end-use. For example, *Nechita *is the best landrace for *genfo, shorba, kinche*; *Tikur gebs *for beverage; *Temej *for *kolo; *whereas *Enat gebs, Sene gebs *and *Meher gebs *are preferred for *injera*. These preferences are related to taste, color, visual appeal, water absorption (volume of product) and storability. According to farmers, *Nechita *is whiter than most landraces, and its food products have visual appeal and appetizing. It has good water absorption capacity, which gives a higher volume of product (e.g. *genfo *and *kinche*). Similarly, the suitability of landraces like *Enat gebs *and *Meher gebs *for their *injera *making quality is related to their water absorption capacity, fermentation process, baking quality, taste and storability of the resulting *injera*. The farmers consider the *injera *from these landraces as tasty and filling, soft, fluffy, spongy with good and well distributed 'eyes'. It stores longer and does not dry quickly or develop mold. The *injera *is usually consumed within four days but it could last upto seven days. *Temej *is the choice for *kolo*. It is hulless and easy to process as it does not require the tedious dehulling process using mortar and pestle, and winnowing before roasting. The roasted grain is tasty and easy to chew. Thus, farmers make all possible efforts to maintain various landraces suitable for specific end-use qualities by planting a landrace in mixtures with other landraces or allotting specific plots of land. It is common to observe mixed (intercropped) planting of many different types in the same field for various other reasons but mainly as buffer for risk aversions. The farmers also reported that mixing improves competition of landraces resulting in a better yield compared with pure stand of either. This may be attributed to the reduction of pests and disease damage, and compensation effects that result from between-plant differences and competition [43]. The practices of mixed planting and maintaining landraces on the basis of distinct functional attributes provide a chance for a low yielding type co-existing with high yielding types, which in turn maintain genetic diversity [7,29]. For example, despite being low yielding, *Temej *is maintained by farmers for its suitability for *kolo*. It is worth noting that preference is dictated by the availability of choice, which in turn is affected by a number of factors including rainfall, soil fertility, plot area and other environmental constraints.

### Genetic erosion

Maintenance of various landraces is driven not only by end-use qualities but also by the rainfall condition of the season, soil type and fertility, maturity, frost tolerance, yield and other factors. Consequently, although farmers' practices enabled on-farm conservation of many landraces, some high-yielding late types like *Enat gebs *and *Ginbote*, which are mainly planted in May or early June, are being selected against because of moisture stress and soil degradation. These landraces are high yielding and are preferred by farmers for many end-use qualities but they require fertile soils and good moisture. The farmers also claimed that the produce and food products from late type landraces last long ("*bereket alew*"). The fertility of the soil is poor and natural resource degradation is the main problem in North Eastern Ethiopia [44-47]. The rainfall is also less reliable and recent climate change data indicated a late start and early cessation of the rainfall for *meher *season [48]. The lack of moisture in May to plant *Ginbote *and *Enat gebs *has shifted the preference to relatively earlier types like *Sene gebs *and *Ehil zer*. In *Wag Hemra *zone with a short *meher *season, farmers have very limited choice other than growing *Tsebel *and *Zibna*, which are early maturing landraces. Similarly, farmers plant early maturing types if the *belg *rain starts late. Generally, farmers are turning more on growing early maturing types that produce low but relatively stable yield. Hence, some of the preferred landraces are being or likely to be marginalized or pushed out of the production system, and this might lead to genetic erosion. Consequently, quality dishes and beverages prepared from the preferred landraces will be compromised with less suitable ones.

It was also reported that the household land holding for barley production has become smaller over the years to produce enough grain due to land fragmentation resulting from population pressure. Consequently, farmers are cutting on some less important ("luxury") dishes and beverages. For example, *tela *is not prepared as frequent as it used to be in 20-30 years time. Hence, landraces suitable for such types of products will ultimately become less important in the maintenance and management of diverse landraces. It may be worth mentioning that no improved barley varieties were grown by the farmers during the study. Thus, improved varieties are not a threat for the loss of landraces. IBC has been implementing on-farm conservation and enhancement of landraces for sorghum in *S.Wello *[49]. The goal is encouraging farmers to continue in maintaining landraces, which enables landraces to evolve for various traits in their area of adaptation. Encouraging results have been achieved with 111 landraces planted as mixtures in farmers' field every year in *Kalu *district [49]. Similarly, such on-farm conservation and enhancement may be relevant for barley landraces conservation in the study area. But the approach requires a sustainable system that benefits participating farmers to guarantee the continuity of the system and provides viable conservation outcomes. On-farm conservation efforts need to be linked to their use to ensure the long survival of crop landraces [13].

## Summary and Conclusions

Barley, as a food and feed grain, is important to the livelihood of farmers in North Eastern Ethiopia. Information on the maintenance of barley landraces and their utilization for food is valuable to design and plan landraces conservation strategies as well as to improve food utilization of barley. The study has shown various reasons for the maintenance of diverse landraces as well as their different food uses, while highlighting the possible erosion of some preferred (culinary qualities) landraces. Incidentally in Ethiopia, most of the food barley varieties released nationally for wide adaptation and those released at state or zone levels for specific adaptation are developed based on yield performance. In the light of this, there is a need to incorporate grain quality as an added objective in barley breeding programs, as well as preserving and improving late maturing landraces.

On suitability for foods and beverages, there is a need for research on the barley quality traits required for the different dishes under different processing conditions including why some landraces are suitable for a specific food product. Some area-specific variations were observed in the way barley foods and beverages were processed. The effects of processing variations on the nutritive content of the barley foods and beverages have not been investigated, and how they par with processing methods in other barley growing areas is not known. The main limitation consistently mentioned by the farmers interviewed in the study with regards to food preparation from barley was that the manual processing of the grain (i.e. dehulling, grinding, sieving, roasting) is very tiresome and time consuming. The manual processing and removal of the hull have also an acknowledged wastage of some grains and it is also likely to have a negative effect on the nutritive value. It is important to investigate how to adapt for Ethiopia the various studies conducted elsewhere on barley as a food with a view to mechanizing many labour-intensive manual operations with simple utensils to maximize the benefits of barley as a food. Moreover, various spices and sauces are used with the different barley dishes. Although these ingredients or additives are known to improve taste, no information is available on their effects on the nutritive values of each dish. There are many nutritional and health benefits of barley, and exploring these for the various Ethiopian traditional foods and beverages could contribute to food security of the country.

## Abbreviations

CSA: Central Statistics Authority, Ethiopia; IBC: Institute of Biodiversity Conservation, Ethiopia.

## Competing interests

The authors declare that they have no competing interests.

## Authors' contributions

HS carried out the field study and wrote the manuscript. PS was involved in revising the manuscript critically for important intellectual content for it to have the present form. All authors read and approved the final manuscript.
